# Non-pharmacological interventions for the treatment of post-intensive care family syndrome in caregivers: A systematic review and meta-analysis

**DOI:** 10.1097/MD.0000000000049538

**Published:** 2026-07-03

**Authors:** Laura Pilar de Paz-Montón, Juan Manuel Carmona-Torres, José Alberto Laredo-Aguilera, Noelia Martín-Espinosa

**Affiliations:** a Faculty of Physiotherapy and Nursing, University of Castilla-La Mancha, Toledo, Spain; b Multidisciplinary Research Group in Care (IMCU), University of Castilla-La Mancha, Toledo, Spain; c Institute of Health Research of Castilla-La Mancha (IDISCAM), Toledo, Spain.

**Keywords:** adults, caregivers, family, post-intensive care syndrome, therapy

## Abstract

**Background:**

Family members of intensive care unit patients are at risk of post-intensive care syndrome-family (PICS-F), including anxiety, depression, post-traumatic stress disorder (PTSD), and reduced quality of life. Non-pharmacological interventions such as counseling, education, and support programs may help, but evidence is limited. Evaluating these interventions is essential to guide tailored strategies that improve caregiver well-being. This systematic review aimed to evaluate the effectiveness of different non-pharmacological interventions for improving post-intensive care family symptoms in family members and caregivers of adult patients.

**Methods:**

A systematic review was conducted according to the Preferred Reporting Items for Systematic Reviews and Meta-Analyses guidelines. Searches were performed in PubMed, Scopus, Web of Science, and ClinicalTrials.gov. Randomized controlled trials (RCTs) evaluating non-pharmacological interventions in adult caregivers of critically ill patients were included. Variables such as anxiety, depression, PTSD, and quality of life were analyzed. Risk of bias was assessed using the Cochrane RoB-2 tool.

**Results:**

Seven RCTs involving 1026 participants, predominantly women, were included. Most interventions did not demonstrate statistically significant improvements in PICS-F symptoms. However, 2 multicomponent interventions that combined structured follow-up, personalized support, and active professional involvement showed significant reductions in anxiety, depression, and PTSD symptoms, as well as improvements in quality of life. In contrast, brief, standardized, or self-guided interventions without sustained professional support were generally ineffective.

**Conclusion:**

Most non-pharmacological interventions evaluated did not show consistent efficacy in reducing PICS-F symptoms. Interventions incorporating longitudinal follow-up, personalized emotional support, and multicomponent approaches appeared to provide greater benefit. Further high-quality RCTs using standardized diagnostic and outcome measures are needed to establish robust therapeutic recommendations.

## 1. Introduction

The intensive care unit (ICU) is a highly specialized healthcare environment designed to provide advanced life support and continuous monitoring to critically ill patients, with the aim of improving outcomes in complex clinical situations. However, the ICU also generates a significant negative impact not only on patients but also on their family members or primary caregivers.^[1]^

Among these adverse effects, special attention has been given to the so-called post-intensive care syndrome, defined as a set of physical, psychological, and cognitive impairments affecting patients who have been admitted to an ICU. Furthermore, the consequences of ICU admission can also affect patients’ relatives or primary caregivers.^[2]^

Post-intensive care syndrome-family (PICS-F) refers to a condition affecting the primary caregiver (whether a family member or not) of a patient who has been admitted to an ICU, manifesting as physical, psychological, or social disturbances.^[3,4]^ Its prevalence ranges from 20% to 40%.^[5]^

The main risk factors for developing this syndrome include personal variables (low educational level, taking a leading role in clinical decision-making during the ICU stay, age, female gender, unemployment, or having recently lost a loved one or witnessed a severe health crisis), healthcare-related factors (poor communication with healthcare staff, lack of information, and restricted visitation), and patient-related factors (severity of illness, delirium, mechanical ventilation, and among others).^[6,7]^

The main physical manifestations of PICS-F are fatigue, sleep disturbances, and general decline. Psychological consequences include emotional distress, anxiety, depression, and post-traumatic stress disorder (PTSD). In some cases, anxiety and depression may worsen, leading to hospitalization.^[6,8,9]^ On a social level, financial insecurity, deterioration of social relationships, and caregiver burden are observed.^[10]^ These physical, psychological, and social consequences of PICS-F not only affect family members on an individual level but also lead to increased healthcare costs, greater use of health services, and more frequent work absenteeism, thus having a significant impact on healthcare systems and the global economy.^[11,12]^

Several recommendations exist to prevent PICS-F. The most relevant include reducing the burden on family members or primary caregivers – namely, allowing flexible presence with the patient and adjusting their caregiving role, which contributes to lower rates of depression and anxiety; fostering effective communication between family members and healthcare staff, including accessible and up-to-date information about the patient’s clinical status. It is also beneficial to involve nurses and/or social workers trained to manage these situations.^[6,9,10]^ Moreover, allowing family members to participate in patient care during the ICU stay can help them feel useful and reduce anxiety levels.^[6]^

Currently, there are no universally validated questionnaires or scales for the evaluation or diagnosis of PICS-F. However, commonly used tools for assessing psychological symptoms include the Hospital Anxiety and Depression Scale (HADS) for anxiety and depression, the Impact of Event Scale-Revised (IES-R/IES‑6) for PTSD, and the Zarit Burden Interview for caregiver burden.^[7]^

Regarding interventions for its treatment, key strategies include providing accurate and timely information and communication about the patient’s condition in the ICU, the use of diaries to record ICU events for later review, follow-up after discharge, and bereavement support in the case of a patient’s death.^[7]^

Nevertheless, there is currently no consensus on the effectiveness of these interventions or the specific conditions under which they should be applied. For this reason, it is necessary to critically assess the efficacy of available interventions.

To date, no updated systematic reviews have analyzed the impact of non-pharmacological therapies or interventions in treating PICS-F. Therefore, the objective of this systematic review was to identify and analyze different non-pharmacological interventions aimed at treating PICS-F in relatives and primary caregivers of adult patients and to evaluate their effectiveness in improving physical, psychological, and social outcomes.

The hypothesis proposed was that applying interventions for PICS-F would improve its associated symptoms.

## 2. Methods

### 2.1. Design and sources of information

A systematic review was conducted following the guidelines of the Preferred Reporting Items for Systematic Reviews and Meta-Analyses (PRISMA) statement.

This systematic review was registered in International Prospective Register of Ongoing Systematic Reviews under registration number CRD420251075110.^[13]^

### 2.2. Research question

The research was conducted using the Population, Intervention, Comparison, and Outcomes (PICO) framework, as shown in Table 1.

**Table 1:** Research question in PICO format.

Population (P)	Intervention (I)	Comparator (C)	Results (O)
Family members and/or caregivers of adult patients admitted to the ICU.	Educational, psychological, informational, or practical interventions or therapies to treat familial post-intensive care syndrome.	Family members and/or caregivers of patients admitted to the ICU who do not receive any intervention or who follow the usual care established in the ICU.	Reduction of psychological, physical, and/or social symptoms.

Own elaboration.

ICU = intensive care unit, PICO = Population, Intervention, Comparison, and Outcomes.

The clinical question was as follows: Are educational, psychological, informative, or practical interventions or therapies effective in treating PICS-F?

To answer this, a search was conducted in the following databases: PubMed, Web of Science, Scopus, and ClinicalTrials.gov between April 1 and July 31, 2025. The search strategy is detailed in Table 2.

**Table 2:** Search strategy used for each database.

Database	Search strategy
PubMed	(“Intensive Care Units”[Mesh] OR “Critical Illness”[Mesh] OR ICU* OR “critical care”) AND (family* OR caregiver* OR relative* OR carer*) AND (“post-intensive care syndrome” OR PICS OR “post ICU” OR “psychological distress” OR anxiety OR depression OR PTSD) AND (intervention* OR program* OR therap* OR support* OR education* OR counseling)
Web of Science	
Scopus	
ClinicalTrials.gov	(“Post intensive care family syndrome”)

Own elaboration.

ICU = intensive care unit, PICS = post-intensive care syndrome.

### 2.3. Inclusion and exclusion criteria

The inclusion criteria for this systematic review were as follows: randomized controlled trials (RCTs) with a control group (CG); family members and/or caregivers with PICS-F; and studies analyzing educational, psychological, informative, or practical interventions or therapies aimed at improving the analyzed variables.

The exclusion criteria were as follows: family members of pediatric patients diagnosed with PICS-F; studies evaluating the efficacy of treatment for PICS in patients; and studies without measurable outcomes.

This systematic review included only RCTs that evaluated various therapies or interventions to address PICS-F. This selection was made to ensure the highest level of scientific evidence and methodological rigor.

### 2.4. Study selection process

The study selection process was conducted collaboratively by LPPM and NME, following the established inclusion and exclusion criteria.

Citations were organized and duplicates removed using the Mendeley reference manager. After duplicate removal, titles and abstracts were reviewed to identify studies for full-text evaluation.

Following a full-text review, the final selection of studies to be included in the qualitative and quantitative synthesis was made. In cases where discrepancies arose during the selection process, discussions were held between LPPM and NME and, if consensus was not reached, a third author, JALA, was consulted to resolve disagreements and reach a consensus.

### 2.5. Quality assessment of the studies

**Figure 1.**
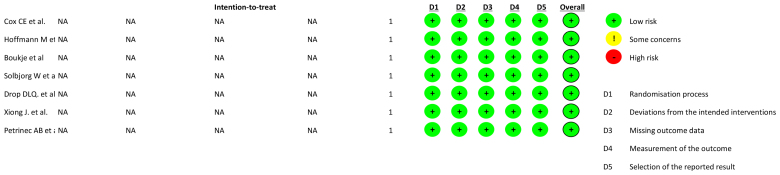
Application of the RoB-2 tool for randomized clinical trials. NA = not available.

To assess the quality of the selected studies and detect bias, the Cochrane RoB-2 tool for RCTs was used.^[14]^ The scores for each study are presented in Figure 1.

The RoB-2 tool consists of 5 domains that evaluate: risk of bias in the randomization process; deviations from intended interventions; missing outcome data; measurement of the outcome variables; and selection of the reported results. Each domain contains 22 items with possible responses of Yes, Probably Yes, Probably No, No, Not Applicable (N/A), and Not Reported (N/R).

For each domain, risk of bias was calculated and categorized as low, high, or concerning. The overall risk of bias was determined as follows: if all domains had low risk, the overall risk was categorized as low; if any domain had a concerning risk, the overall risk was categorized as concerning; and if any domain had a high risk or if several domains were concerning, the overall risk was categorized as high.

### 2.6. Data extraction

Data extraction was carried out by LPPM and JMCT. The results were extracted using Mendeley, manually classified, and thoroughly reviewed. The following information was extracted from each article: first author, year, and country; study design; participant characteristics, including sample size, mean age, gender, and selection process; study intervention; variables studied (including the measurement tools used); and main outcomes, outcome assessment, and limitations.

### 2.7. Data analysis

A narrative synthesis was performed for each study included in this review. The influence of interventions on PICS-F was analyzed before and after the intervention.

For the quantitative synthesis, random-effects meta-analyses were performed using RevMan 5.4 (The Cochrane Collaboration). The analyses compared the scores obtained from the different instruments used to assess PICS-F symptoms in order to determine the effectiveness of the interventions. Effect sizes were calculated based on post-intervention outcomes reported by the included studies. Statistical heterogeneity was assessed using the *I*^2^ statistic and was interpreted as follows: *I*^2^ ≤ 25% indicated low heterogeneity, 26% to 50% indicated moderate heterogeneity, and ≥51% indicated high heterogeneity. Statistical significance was set at *P* < .05.

## 3. Results

### 3.1. Study selection and characteristics

Four databases were consulted, and after applying the previously defined search strategy, a total of 832 articles were retrieved. Of these, 397 were duplicates, resulting in 435 unique articles. After screening titles and abstracts, 12 full-text articles were assessed, and only 7 RCTs published between 2018 and 2025 were selected for analysis.

**Figure 2.**
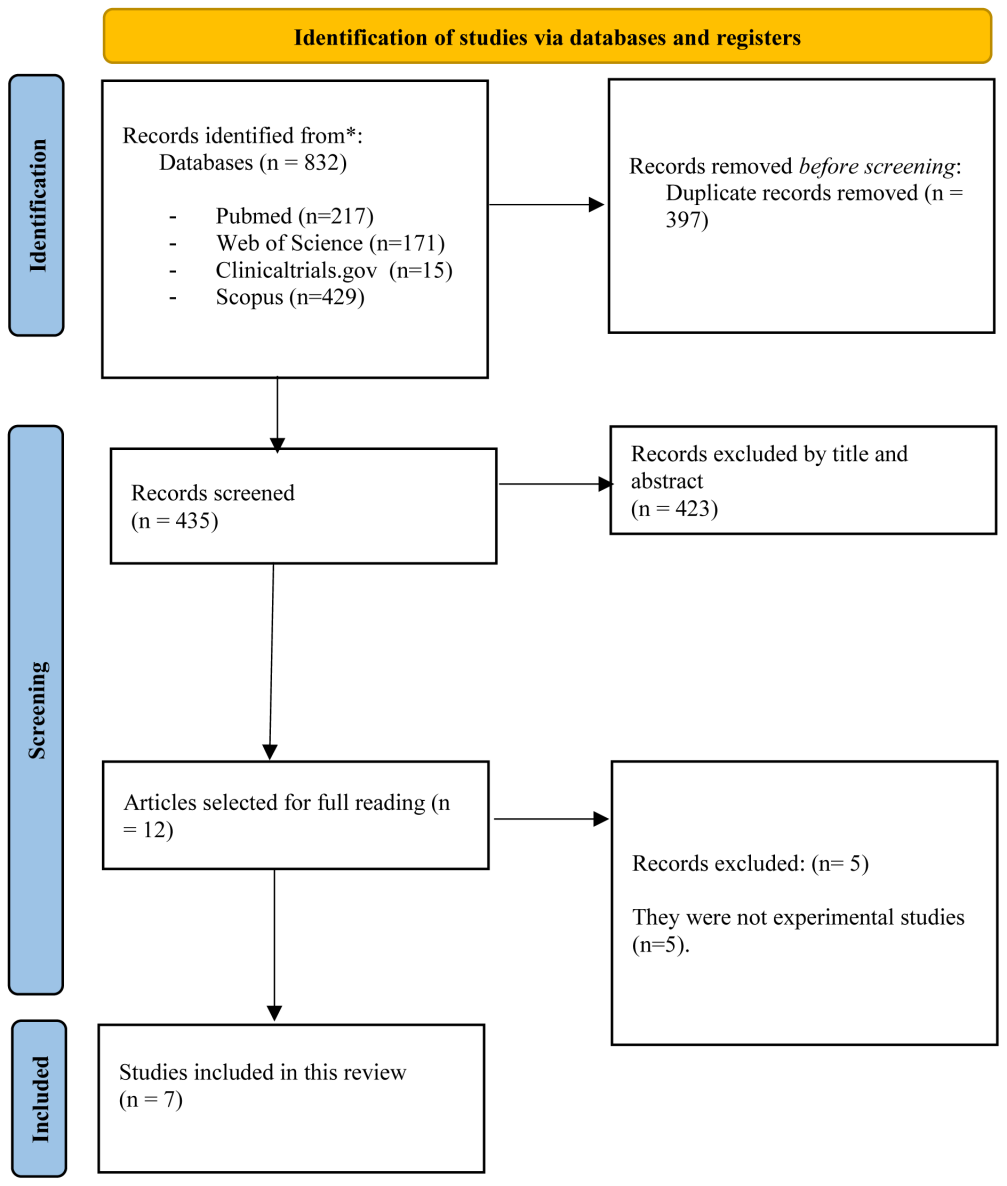
Flowchart for study selection.

In total, the included studies involved 1026 participants, of whom 439 were assigned to the intervention group (IG) and 587 to the control or comparison group. Figure 2 shows the PRISMA flow diagram developed for the selection process in this systematic review.

All studies included adult caregivers and/or family members of patients admitted to ICUs, predominantly women aged between 46 and 63 years. The most commonly evaluated variables were symptoms of anxiety and depression, PTSD, and health-related quality of life.

In all studies, the interventions adhered to established ethical standards and received approval from the corresponding ethics committees.

Table 3 presents the data analysis and main characteristics of the studies included in this systematic review.

**Table 3:** Data analysis and main characteristics of the studies.

Study	Design	Participants	Interventions	Evaluation and results	Limitations
Cox et al,^[15]^ USA.	RCT	N = 86 IG: 39 (33 W) CG: 47 (36 W)	IG: Telephone-based coping skills training for 6 wk, including relaxation, communication, cognitive restructuring, and activity management.	Anxiety and depression: HADS PTSD: IES-R	Losses to follow-up in both groups.
		A: 51.5	CG: Educational web-based ICU information program.	No significant differences after intervention and between groups.	
				HADS: T1: MD = 1.3; 95% CI: (−0.9, 3.7); *P* = .24	
				IES-R: T1: MD = 3.1; 95% CI: (−1.9, 8.1); *P* = .22 T2: MD = 3.6; 95% CI: (−2.7, 10.0); *P* = .26	
Hoffmann et al,^[16]^ Austria and Switzerland.	RCT	N = 89 IG: 46 (30 W) CG: 43 (29 W)	IG: Online ICU educational platform with expert chats, videos, and testimonials.	IES-R HADS	High losses to follow-up.
		A: 47.3	CG: Basic ICU information webpage.	No significant improvements in PICS-F symptoms.	
				IES: T1: MD = −0.2; 95% CI: (−6.86, 6.46); *P* = .892	
				HADS: T1: MD = 4.12; 95% CI: (2.2, 5.99); *P* = .552	
Petrinec et al,^[17]^ USA.	RCT	N = 60 IG: 30 (24 W) CG: 30 (23 W)	IG: Self-care mental health app (Sanvello), including mindfulness and emotional management modules.	HADS PTSD: PCL-5 HRQoL: SF-12	Small sample size.
		A: 46.4	CG: No intervention.	No significant improvements after intervention.	
				HADS: T1: MD = −0.58; 95% CI: (−4.26, 3.10); *P* = .10 T2: MD = 0.44; 95% CI: (−3.30, 4.18); *P* = .50	
				PCL-5: T1: MD = 4.93; 95% CI: (−8.29, 18.15); *P* = .20 T2: MD = 2.89; 95% CI: (−9.37, 15.15); *P* = .20	
				SF-12: T2: MD = −0.28; 95% CI: (−9.66, 9.10); *P* = .20	
Dijkstra et al,^[18]^ Netherlands.	RCT	N = 306 IG: 73 (34 W) CG: 233 (107 W)	IG: Structured family participation program led by nurses and physicians in ICU patient care activities.	HADS IES-R	Need for longer follow-up and additional measurements.
		A: 62.5	CG: No intervention.	No significant improvements after intervention.	
				HADS: T1: MD = −072; 95% CI: (0.46, 1.13); *P* = .39	
				IES-R: T1: MD = 0.94; 95% CI: (0.78, 1.14); *P* > .05	
Watland et al,^[19]^ Norway.	RCT	N = 196 IG: 101 (68 W) CG: 95 (59 W)	IG: 4-step caregiver pathway intervention including nurse support and post-discharge follow-up.	HADS IES-R HRQoL: SF-12	Limited eligibility information. Up to 3 caregivers included per patient.
		A: 46.8	CG: Standard care.	Significant improvements in anxiety, PTSD symptoms, and HRQoL.	
				HADS: T1: MD = −1.85; 95% CI: (−3.45, −0.30); *P* = .011*	
				IES-R: T1: MD = −8.2; 95% CI: (−14.2, −2.2); *P* = .008*	
				HRQoL: T1: MD = 2.4; 95% CI: (0.4, 4.3); *P* = .017*	
Drop et al,^[20]^ Netherlands.	RCT	N = 189 IG: 100 (53 W) CG: 89 (48 W)	IG: Virtual reality ICU educational intervention plus standard care.	HADS IES-R HRQoL: SF-36	High attrition during follow-up.
		A: 47.5	CG: Standard care.	No significant improvements in PICS-F symptoms.	
				HADS: T2: MD = 0.215; 95% CI: (−0.535, −0.96); *P* = .42	
				IES-R: T2: MD = −7.32; 95% CI: (−9.13, −5.51); *P* < .06	
				SF-36: T2: MD = −3.27; 95% CI: (−5.05, −2.56); *P* = .38	
Xiong et al,^[21]^ China.	RCT	N = 100 IG: 50 (32 W) CG: 50 (29 W)	IG: Multicomponent online intervention with emotional and professional support from ICU admission to 1 mo post-discharge.	HADS IES-R SF-36	Single-center study. Potential participant bias.
		A: 50	CG: No intervention.	Significant reductions in anxiety, depression, and PTSD symptoms and improvement in quality of life.	
				HADS: T1: MD = 1.92; 95% CI: (1.49, 2.35); *P* < .001** T2: MD = 1.66; 95% CI: (1.29, 2.03); *P* < .001** T3: MD = 3.98; 95% CI: (3.62, 4.34); *P* < .001**	
				IES-R: T2: MD = 6.45; 95% CI: (19.50, 25.38); *P* < .001**	
				HRQoL: T2: MD = 31.16; 95% CI: (21.35, 40.98); *P* < .001**	

A = mean age, CG = control group, CI = confidence interval, HADS = Hospital Anxiety and Depression Scale, HRQoL = health-related quality of life, ICU = intensive care unit, IES = Impact of Event Scale, IES-R = Impact of Event Scale-Revised, IG = intervention group, MD = mean difference, PCL-5 = 20-item post-traumatic stress disorder (PTSD) checklist for the Diagnostic and Statistical Manual of Mental Disorders PCL-5 instrument, PICS-F = post-intensive care syndrome-family, PTSD = post-traumatic stress disorder, RCT = randomized controlled trial, SF-12 = Short Form 12-Item Health Survey, SF-36 = Medical Outcomes Study 36-Item Short Form, T0 = pre-intervention measure, T1/2/3 = post-intervention measures, W = women.

* *P* < .05.

** *P* < .001.

### 3.2. Effectiveness of the interventions

Of the 7 studies analyzed, 5 did not show statistically significant differences in PICS-F symptoms between the intervention and CGs.^[15–18,20]^ Only 2 studies demonstrated statistically significant improvements in the analyzed variables following the intervention.^[19,21]^

The studies evaluated variables such as anxiety, depression, PTSD, and quality of life.

### 3.3. Informative and educational interventions

Four studies based their interventions on educational content delivered through digital tools, focusing on both teaching coping strategies and providing specific information about ICU operations to help caregivers become familiar with the hospital environment.^[15,16,20,21]^ The study by Xiong et al^[21]^ was the only one to show positive results in reducing anxiety, depression, PTSD, and improving quality of life. This study stood out for its longitudinal and intensive structure, divided into 3 phases (ICU admission, ward transition, and post-discharge), and for its direct and ongoing interaction with a professional team (doctors, nurses, and students) who provided both personalized information and active emotional support. This combination of extended duration, professional accompaniment, and synchronous multicomponent design likely explains its superior clinical effectiveness compared with other interventions focused solely on standardized informational content or self-guided digital resources without ongoing professional interaction.

### 3.4. Psychological or digital self-care interventions

Two studies^[17,19]^ developed psychological interventions based on self-care and emotional self-management. Petrinec et al^[17]^ used a mental health app (Sanvello) with structured modules that caregivers completed autonomously without professional contact and found no significant improvements. In contrast, Watland et al^[19]^ reported statistically significant improvements in anxiety reduction, PTSD symptoms, and quality of life. A possible key to success was the structured support provided by nursing staff, which was implemented in 4 phases: assessment of concerns and needs with active support during ICU admission; delivery of personalized informational materials and useful contacts at ICU discharge; proactive text message contact after hospital discharge to offer follow-up; and a follow-up phone interview by nurses during the first 3 months post-discharge to evaluate caregiver adjustment and offer intervention if necessary. This structure enabled a continuous, proactive, and empathetic presence by the nursing team, extending beyond hospitalization and likely contributing to the intervention’s effectiveness.

### 3.5. Participatory or practical interventions

Dijkstra et al^[18]^ evaluated a participatory intervention focused on the active involvement of family members in the care of ICU patients through a structured family participation program. The intervention allowed family members to collaborate with professionals in activities such as communicating with the patient, assisting with mobilization, respiratory exercises, nutrition, daily activities, and entertainment, with the goal of making them feel useful and reducing feelings of helplessness.

However, the intervention did not include explicit emotional or psychological support components, such as active listening sessions, stress-coping techniques, individualized professional support, or strategies targeting the emotional impact of hospitalization. In other words, the intervention focused exclusively on practical and functional aspects, without directly addressing the caregiver’s emotional well-being or psychological needs.

### 3.6. Meta-analysis of psychological outcomes

A quantitative synthesis was performed for the most frequently reported and comparable outcomes across studies: anxiety and depression assessed with the HADS, and post-traumatic stress symptoms assessed with the IES-R.

**Figure 3.**
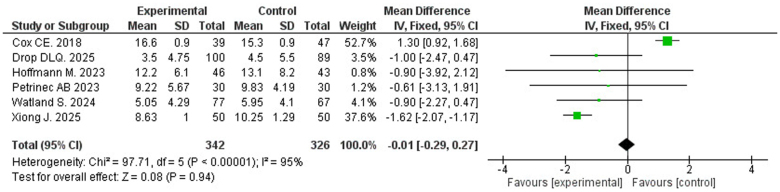
Forest plot of HADS. CI = confidence interval, HADS = Hospital Anxiety and Depression Scale, SD = standard deviation.

For HADS outcomes, 6 studies involving 668 participants in the IG and 646 participants in the CG were included. The pooled analysis showed no significant effect of the interventions on anxiety and depression symptoms compared with control conditions (MD = −0.01, 95% confidence interval: −0.29 to 0.27; *P* = .94). A high level of heterogeneity was observed among studies (*I*^2^ = 95%), indicating substantial variability in intervention characteristics and study populations (Fig. 3).

**Figure 4.**
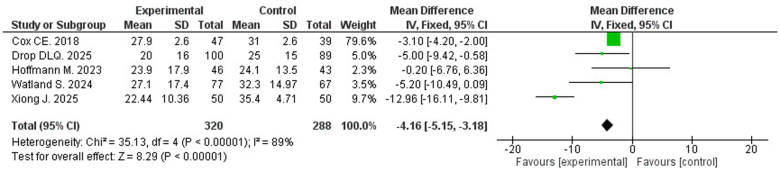
Forest plot of IES-R. IES-R = Impact of Event Scale-Revised.

For PTSD symptoms assessed with the IES-R, 5 studies involving 320 participants in the IG and 288 participants in the CG were included. The pooled analysis demonstrated a significant reduction in PTSD symptoms favoring the IGs (MD = −4.16, 95% confidence interval: −5.15 to −3.18; *P* < .00001). However, heterogeneity was also high (*I*^2^ = 89%; Fig. 4).

Overall, the meta-analysis suggests that non-pharmacologicalinterventions may contribute to reducing PTSD symptoms among family caregivers of ICU patients, whereas no significant pooled effect was observed for anxiety and depression outcomes measured using the HADS.

### 3.7. Risk of bias

The 7 included studies were assessed for risk of bias using the Cochrane RoB-2 tool for RCTs; the results are shown in Figure 1.

All 7 studies were rated as having a low risk of bias.^[15–21]^

## 4. Discussion

PICS-F has shown great relevance in the clinical field due to its long-term impact on the physical, psychological, and emotional health and quality of life of caregivers of critically ill patients.^[5,7]^ Moreover, societal changes have contributed to an increase in this syndrome, which has generated growing scientific interest and has been studied more frequently, with protocols for future RCTs of interventions for PICS-F treatment already established.^[22,23]^ However, despite this increased attention, interventions designed to mitigate its effects still present contradictory results, and experimental studies testing them are scarce.

This systematic review shows that most of the analyzed interventions^[15–18,20]^ did not produce statistically significant improvements in PICS-F symptoms, aligning with previous systematic reviews evaluating family-centered interventions in intensive care settings.^[24,25]^ Kentish-Barnes et al^[24]^ reported that interventions based exclusively on information delivery or isolated communication strategies often showed limited effects on psychological outcomes among caregivers. Similarly, Williams et al^[25]^ highlighted that interventions lacking continuity of care and emotional support frequently failed to reduce stress-related symptoms in family members of critically ill patients.

One possible explanation for the limited effectiveness observed in several studies may be the passive nature of many interventions.^[26,27]^ Educational or informational strategies delivered without personalization, emotional support, or longitudinal follow-up may be insufficient to address the complex psychological burden associated with PICS-F.^[26]^ In contrast, interventions that incorporate continuous professional support, individualized communication, and follow-up after ICU discharge appear to achieve better outcomes. These findings suggest that PICS-F may require multidimensional interventions addressing both emotional and practical caregiver needs over time.^[27]^

One possible reason for their lack of efficacy could be attributed to the design and content of the educational interventions proposed, as several focus exclusively on providing information about the ICU and techniques mainly used during admission, without adding emotional support or follow-up by healthcare professionals during the stay and at hospital discharge.^[13,14,18]^ Some authors^[25,28]^ point out that information without emotional support may even increase stress in certain caregivers by raising awareness of the severity of the situation without providing adequate coping tools, potentially intensifying feelings of helplessness, anxiety, and emotional overload.

In contrast, interventions that combine multiple components (follow-up, support, structured communication, and other components) showed improvements in symptoms of anxiety, depression, and PTSD and also enhanced quality of life,^[19,21]^ consistent with other authors who argue that PICS-F interventions should be designed following a concept of therapeutic continuity and should consider the family as a treatment unit.^[26,29]^

Furthermore, it is important to highlight the duration and timing of interventions, as applying them only during the hospital stay seems to be insufficient.^[20]^ The emotional impact of having been involved in an ICU admission manifests over time and intensifies after hospital discharge, suggesting the need to maintain interventions at discharge and during the adaptation and transition process to this new reality.^[29,30]^

Additionally, a common element in studies with unfavorable results is the lack of adaptation of the tested interventions to the characteristics of each caregiver, as standard tools were used without considering the specific context of each person. As the literature emphasizes, educational interventions need to be tailored to the social, cultural, and emotional profile of the caregiver to achieve the desired effect, which may explain the low efficacy due to this lack of adaptation.^[6,31]^

Another important issue is the absence of universally accepted diagnostic criteria and validated instruments specifically designed for PICS-F assessment. Although tools such as the HADS and the IES-R are widely used to evaluate anxiety, depression, and post-traumatic stress symptoms, these instruments were not originally developed for caregivers of ICU patients. Consequently, their ability to fully capture the multidimensional nature of PICS-F may be limited.^[3,10]^ Moreover, variability in outcome measures across studies reduces comparability among interventions and complicates the interpretation of findings. The development and validation of standardized PICS-F-specific assessment tools would improve diagnostic accuracy and strengthen future research in this field.^[32,33]^

Finally, none of the studies included a cost-effectiveness evaluation of the interventions, which is a priority for public health policy design, because PICS-F may lead to increased use of health resources and higher rates of work absenteeism. Cost-effective interventions should be developed to help minimize the impact of PICS-F.^[34,35]^

Overall, the results of this systematic review suggest the need to develop comprehensive, adaptive, and sustained interventions over time, implemented by trained healthcare professionals who recognize the specific clinical framework of PICS-F.

### 4.1. Limitations

This systematic review presents a series of strengths that reinforce the robustness of the findings. First, methodological rigor, as PRISMA guidelines were followed, and the review is registered in International Prospective Register of Ongoing Systematic Reviews, ensuring transparency and reproducibility of results. Additionally, updated randomized clinical trials were included, providing high scientific evidence studies that allow a critical evaluation of the literature with methodological robustness, using the standardized Cochrane Rob-2 tool for risk of bias assessment.

This review has several limitations that should be considered when interpreting the findings. First, the included studies showed substantial heterogeneity in intervention content, duration, delivery format, follow-up periods, and outcome assessment methods, which limited direct comparisons and contributed to the high heterogeneity observed in the meta-analyses. Second, the findings are inherently constrained by the methodological characteristics of the available evidence, including the limited number of RCTs, the scarcity of longitudinal and multicomponent interventions, and the relatively small sample sizes and losses to follow-up reported in some studies. Third, the absence of standardized and validated instruments specifically designed to assess PICS-F may have reduced the comparability and sensitivity of outcome measurements across studies. In addition, publication bias cannot be excluded, as formal assessment methods such as funnel plots were not feasible due to the small number of included studies, although a meta-analysis was performed. Language bias may also be present because only studies published in English were included. This was done because English is the dominant language of international scientific publication. Timeframe bias cannot be ruled out; however, the included studies are recent because no earlier RCTs that met the inclusion criteria for this review were identified.

## 5. Conclusion

This systematic review showed that most of the interventions used in the analyzed studies were not effective in reducing symptoms associated with PICS-F, suggesting that other types of interventions should be tested, based on those that demonstrated significant benefits: multicomponent, with active professional support and follow-up, and maintained after discharge.

To date, there are no studies evaluating PICS-F as a global disorder affecting different spheres of caregivers of ICU-hospitalized patients. It would be necessary to develop validated tools to adapt interventions to the real impact generated by this condition.

The heterogeneity of the studies, the lack of specific diagnostic tools, and the methodological limitations described indicate the need to continue conducting experimental research in this field, testing comprehensive, personalized, evidence-based interventions with follow-up, in order to advance towards a model of healthcare that considers the caregivers of critically ill patients as a figure requiring attention and care.

## Acknowledgments

The authors would like to thank the University of Castilla-La Mancha research support office for their support in the development of this review.

## Author contributions

**Conceptualization:** Laura Pilar de Paz-Montón, José Alberto Laredo-Aguilera.

**Methodology:** Laura Pilar de Paz-Montón, Juan Manuel Carmona-Torres, José Alberto Laredo-Aguilera.

**Formal analysis:** Juan Manuel Carmona-Torres, Noelia Martín-Espinosa.

**Investigation:** Laura Pilar de Paz-Montón, José Alberto Laredo-Aguilera.

**Writing – original draft:** Laura Pilar de Paz-Montón, José Alberto Laredo-Aguilera.

**Writing – review & editing:** Laura Pilar de Paz-Montón, Juan Manuel Carmona-Torres, José Alberto Laredo-Aguilera, Noelia Martín-Espinosa.
